# Clinical analysis of the “small plateau” sign on the flow-volume curve followed by deep learning automated recognition

**DOI:** 10.1186/s12890-021-01733-x

**Published:** 2021-11-09

**Authors:** Yimin Wang, Wenya Chen, Yicong Li, Changzheng Zhang, Lijuan Liang, Ruibo Huang, Jianling Liang, Yi Gao, Jinping Zheng

**Affiliations:** 1 National Center for Respiratory Medicine, State Key Laboratory of Respiratory Disease, National Clinical Research Center for Respiratory Disease, Guangzhou Institute of Respiratory Health, First Affiliated Hospital of Guangzhou Medical University, Yanjiang Road 151, Guangzhou, 510120 Guangdong People’s Republic of China; 2 Tsinghua-Berkeley Shenzhen Institute, Tsinghua University, Shenzhen, 518055 People’s Republic of China; 3 Huawei Cloud BU EI Innovation Laboratory, Huawei Technologies, Shenzhen, 518129 People’s Republic of China

**Keywords:** Airway responsiveness, Deep learning, Flow-volume curve, Pulmonary function test, Small plateau sign

## Abstract

**Background:**

Small plateau (SP) on the flow-volume curve was found in parts of patients with suspected asthma or upper airway abnormalities, but it lacks clear scientific proof. Therefore, we aimed to characterize its clinical features.

**Methods:**

We involved patients by reviewing the bronchoprovocation test (BPT) and bronchodilator test (BDT) completed between October 2017 and October 2020 to assess the characteristics of the sign. Patients who underwent laryngoscopy were assigned to perform spirometry to analyze the relationship of the sign and upper airway abnormalities. SP-Network was developed to recognition of the sign using flow-volume curves.

**Results:**

Of 13,661 BPTs and 8,168 BDTs completed, we labeled 2,123 (15.5%) and 219 (2.7%) patients with the sign, respectively. Among them, there were 1,782 (83.9%) with the negative-BPT and 194 (88.6%) with the negative-BDT. Patients with SP sign had higher median FVC and FEV_1_% predicted (both *P* < .0001). Of 48 patients (16 with and 32 without the sign) who performed laryngoscopy and spirometry, the rate of laryngoscopy-diagnosis upper airway abnormalities in patients with the sign (63%) was higher than those without the sign (31%) (*P* = 0.038). SP-Network achieved an accuracy of 95.2% in the task of automatic recognition of the sign.

**Conclusions:**

SP sign is featured on the flow-volume curve and recognized by the SP-Network model. Patients with the sign are less likely to have airway hyperresponsiveness, automatic visualizing of this sign is helpful for primary care centers where BPT cannot available.

## Background

Spirometry flow-volume curve displays airflow versus volume during maximum forced inspiration and expiration, it can be influenced by various factors [1]. According to international standards, types of ventilatory defects can be inferred from the configuration of the curve, obstructive abnormalities are thought to be a concave shape on the curve, the curve of a restrictive ventilatory defect shows a convex pattern [2]. Furthermore, Miller and Hyatt [3] found that flattening on the inspiratory and/or expiratory phase of the curve could suggest upper airway obstruction. Flow oscillations referred to as a “saw-tooth” sign seen on curves are thought to be an indicator of obstructive sleep apnea [4]. The flow-volume curve display is diversified in patients with different diseases, as the disease progresses, so does the curve configuration.

Li et al. [5] first found a small plateau (SP) in the early phase of expiratory flow (mostly located at the phase from PEF to FEF_50%_). They identified the sign in 228 of 808 patients (28.2%) who completed bronchoprovocation test (BPT), about 196 (86%) of patients had negative-BPT indicating that patients with the sign maybe have a negative trend prevalence of airway hyperresponsiveness (AHR). Moreover, they found that patients with the sign had a prevalence of symptoms due to upper airway abnormalities, including severe cough and hoarseness when complete the methacholine challenge maneuvers, which indicated that the sign may be related to abnormal structure or function of the upper airway. Xie et al. [6] similarly found that patients were more often negative-BPT (95.2%) in a total of 124 subjects with SP sign who performed BPT, they further confirmed that patients with SP sign may less likely to have AHR. BPT is used to evaluate AHR which is most commonly associated with asthma [7]. However, due to a variety of reasons, BPT cannot be available in primary care settings. In addition, the test is time-consuming and usually requires specialists. At present, asthma has a rapid rise in prevalence in low-income and middle-income countries [8]. In high-income countries, it has high rates of exacerbation and re-admissions [9], which has been proved associated with lung function [10]. Some previous studies have focused on finding simple methods to evaluate AHR and diagnose asthma, like questionnaires and baseline spirometry parameters [11, 12]. The identification and application of the SP sign may have crucial value in predicting the absence of AHR to avoid unnecessary testing.

With the availability of portable spirometers in primary care, forced spirometry (FS) has become easily accessible to all levels of healthcare. In clinical practice, however, most non-professionals lack the ability or experience to identify the configuration of the flow-volume curve, which may lead to the need for a simple and effective approach for visual inspection of the curve. The application of artificial intelligence in medicine is rising quickly [13]. Deep learning is a branch of artificial intelligence that could provide a simple and useful tool to automate spirogram recognition [14]. Though it is possible that some simpler computer vision (CV) techniques could also solve the problem, deep learning-based CV methods are able to outperform those traditional CV and signal processing methods in most scenarios [15–19]. The reason behind this is that the deep learning model often encompasses a large parameter space and does not require hand-crafted features but instead automatically learns appropriate features. These characteristics give the deep learning model a stronger capacity to model the task and better generalization abilities compared to traditional methods. Since deep learning has produced promising results in tackling the display of the curve, we hypothesize that the application of deep learning is ideal for the automatic recognition of the SP sign.

In trying to analyze the potential use of the SP sign as a negative marker for AHR in patients with suspected asthma, we retrospectively reviewed the characteristics of the SP sign in BPT. Since FEV_1_ < 60% predicted is a contraindication for BPT, we also reviewed bronchodilator tests (BDTs) to involve patients with severe ventilatory defects. Furthermore, for patients who required laryngoscopy according to specialists’ decision, FS was performed after they have completed the laryngoscopy tests to assess the laryngoscopy-diagnosis upper airway abnormalities of patients with the sign. At last, we leveraged deep learning algorithms to automatically tackle the recognition problem of the SP sign.

## Methods

This study was conducted at the First Affiliated Hospital of Guangzhou Medical University, using BPTs and BDTs data from October 2017 to October 2020 and FS data during the period 8 March 2021 to 26 March 2021. The study was approved by the Ethics Committee of the First Affiliated Hospital of Guangzhou Medical University (approval NO.: 2020124).

### Pulmonary function tests (PFTs)

BPTs and BDTs were performed on MasterScreen-Pneumo (Jaeger, Germany) and QuarkPFT (COSMED, Italy). FS tests were performed by portable spirometer with U-BREATH PF680 (E-linkcare, China). Quality control of pre-and post- PFTs followed by guidelines, baseline spirometry should achieve three acceptable FEV_1_ and FVC measurements [1, 20]. Post-BPT and post-BDT acceptable FEV_1_ and FVC measurements were also needed. A positive-BPT required a 20% fall from the post-diluent baseline FEV_1_ after the last dose of methacholine [21]. A positive-BDT was identified when the percent change from baseline and absolute changes in FEV_1_ and/or FVC values ≥ 12% and ≥ 200 ml [2].

### Study design

**Fig. 1:**
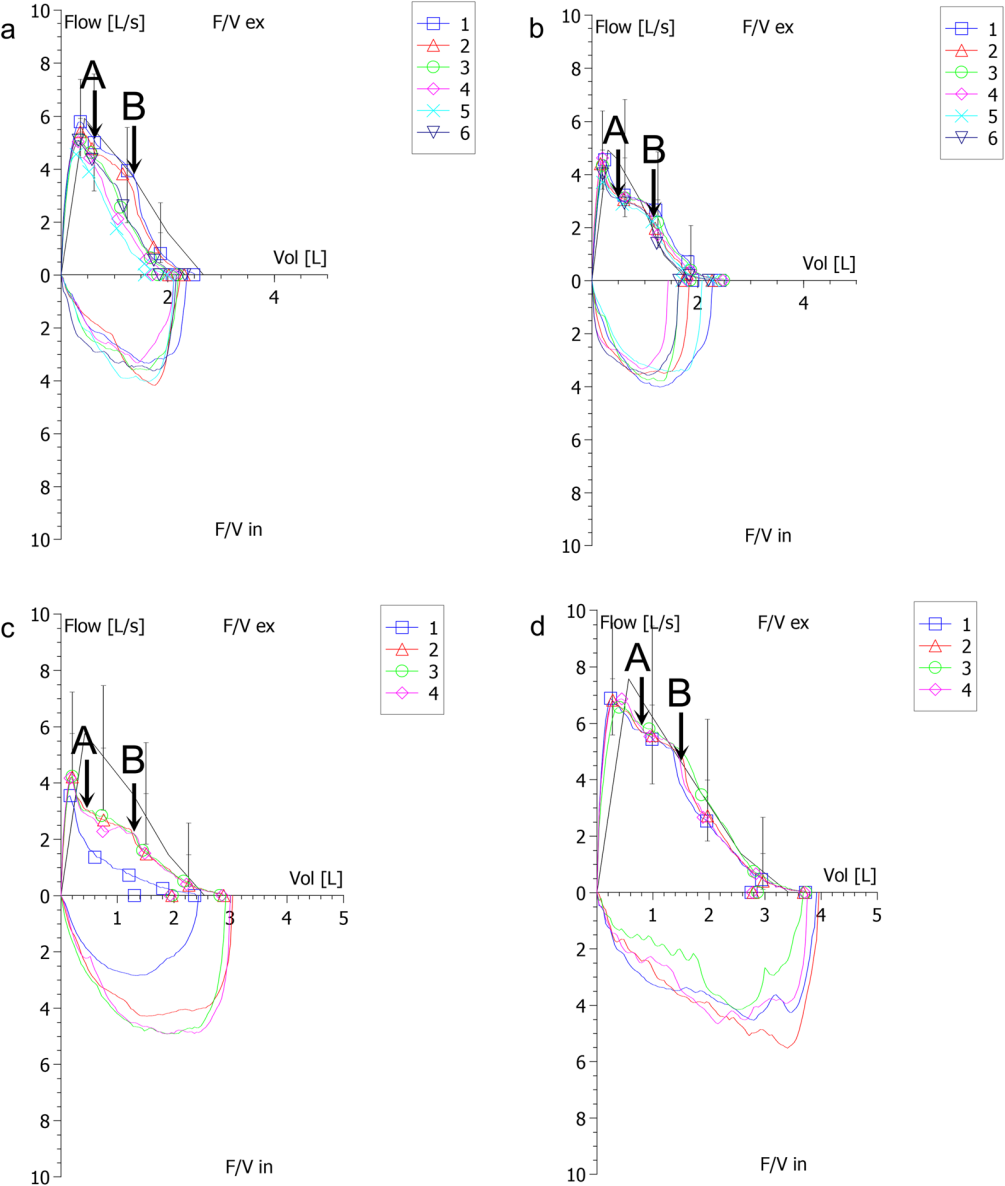
Representative examples of the SP sign in BPTs and BDTs. **a** SP sign in positive BPT. Curve 1 = pre-BPT, SP sign (+); Curve 5 = post-BPT, SP (−). **b** SP sign in negative BPT. Curve 1 = pre-BPT, SP sign (+); Curve 6 = post-BPT, SP sign (+). **c** SP sign in positive BDT; Curve 1 = pre-BDT, SP sign (−); Curve 4 = post-BDT, SP (+). **d** SP sign in negative BDT; Curve 1 = pre-BDT, SP sign (+); Curve 4 = post-BDT, SP sign (+). Point A = the start point of the SP sign; Point B = the end point of the SP sign. SP = small plateau; BPTs = bronchoprovocation tests; BDTs = bronchodilator tests

Four junior pulmonologists determined the SP sign based on a repeatable small plateau on the flow-volume curves. They independently labelled different portions of total curves (never the same) and all have experience in operating and interpreting spirometry for more than 2 years. If they have any doubts, then a senior pulmonologist with experience of more than 18 years would make the final decision. Representative examples of the SP sign were shown in Fig. 1, each BPT or BDT report has at least three flow-volume curves. Point A is the start point of the sign, point B is the endpoint. The data points of A and B were extracted using Engauge Digitizer 11.1 software (M. Mitchell, Engauge Digitizer). Volume (Vol) A/FVC and Flow A/PEF were original (x, y) data of point A; Vol B/FVC and Flow B/PEF were original (x, y) data of point B. (Vol A − Vol B) /FVC and (Flow A − Flow B) /PEF represent the width and height of the sign, respectively. Pre-PFT questionnaires and electronic health records were reviewed by the same authors to identify symptoms and PFT indications. To further evaluate the features of the sign, according to the width or height of the sign, subjects were stratified into four classes, respectively:

- Class 1: (Vol A − Vol B) × 100/FVC ratio or (Flow A − Flow B) × 100/PEF ratio ≤ 10%
- Class 2: (Vol A − Vol B) × 100/FVC ratio or (Flow A − Flow B) × 100/PEF ratio > 10% to ≤ 20%
- Class 3: (Vol A − Vol B) × 100/FVC ratio or (Flow A − Flow B) × 100/PEF ratio > 20% to ≤ 30%
- Class 4: (Vol A − Vol B) × 100/FVC ratio or (Flow A − Flow B) × 100/PEF ratio > 30%

The study randomly selected 50 subjects with the sign from each class to compare the PFT and clinical features. All the electronic health records were reviewed within 6 months of PFTs.

For patients who underwent laryngoscopy according to specialists’ decision in the department of otolaryngology, FS tests were performed after laryngoscopy was completed. Patients who had contraindications for PFTs were excluded according to the Chinese Thoracic Society guidelines [22].

### Development of the model

**Fig. 2:**
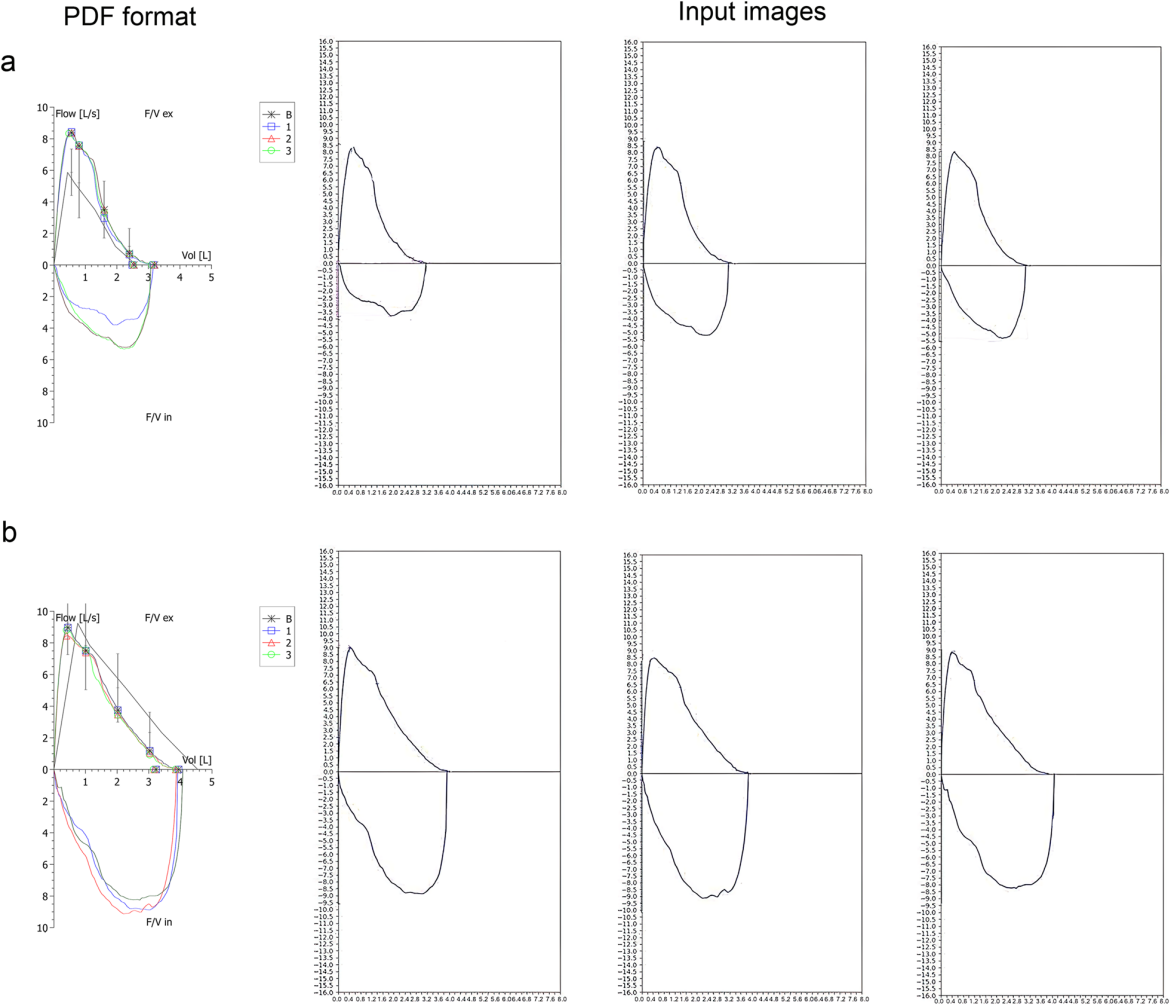
A couple of examples of pdf and input images. Inputs to the model were images extracted from collected PFT reports in pdf format. **a** Flow-volume curves of a woman (aged 64 years, height 162.8 cm) with pdf on the left, after pre-processing of curves by the model were used as input images on the right; **b** Flow-volume curves of a man (aged 25 years, height 165.5 cm). PFT = pulmonary function test

3,453 (1953 SP sign positive and 1500 negative samples) and 374 samples (95 positive and 279 negative samples) were used for training and testing the model, respectively. 294 positive samples could not be used due to the model failure to extract the image. Inputs to the model were images extracted from collected PFT reports in pdf. format, to be specific, flow-volume curves were saved as vector graphics and could be extracted by developing Python scripts. All extracted curves were further processed to have a pixel size of 600 ∗ 1200 by using the OpenCV-Python package. A couple of examples of pdf and curve images can be found in Fig. 2. Each PFT report must have at least three flow-volume curves, only when repeatable (at least three curves) detecting of the SP sign, then the model would output the presence of the sign. In this work, the recognition of the SP sign was formulated as a detection task. To this end, we proposed SP Network (SP-Net), a Faster R-CNN [18] alike object detection model.

**Fig. 3:**
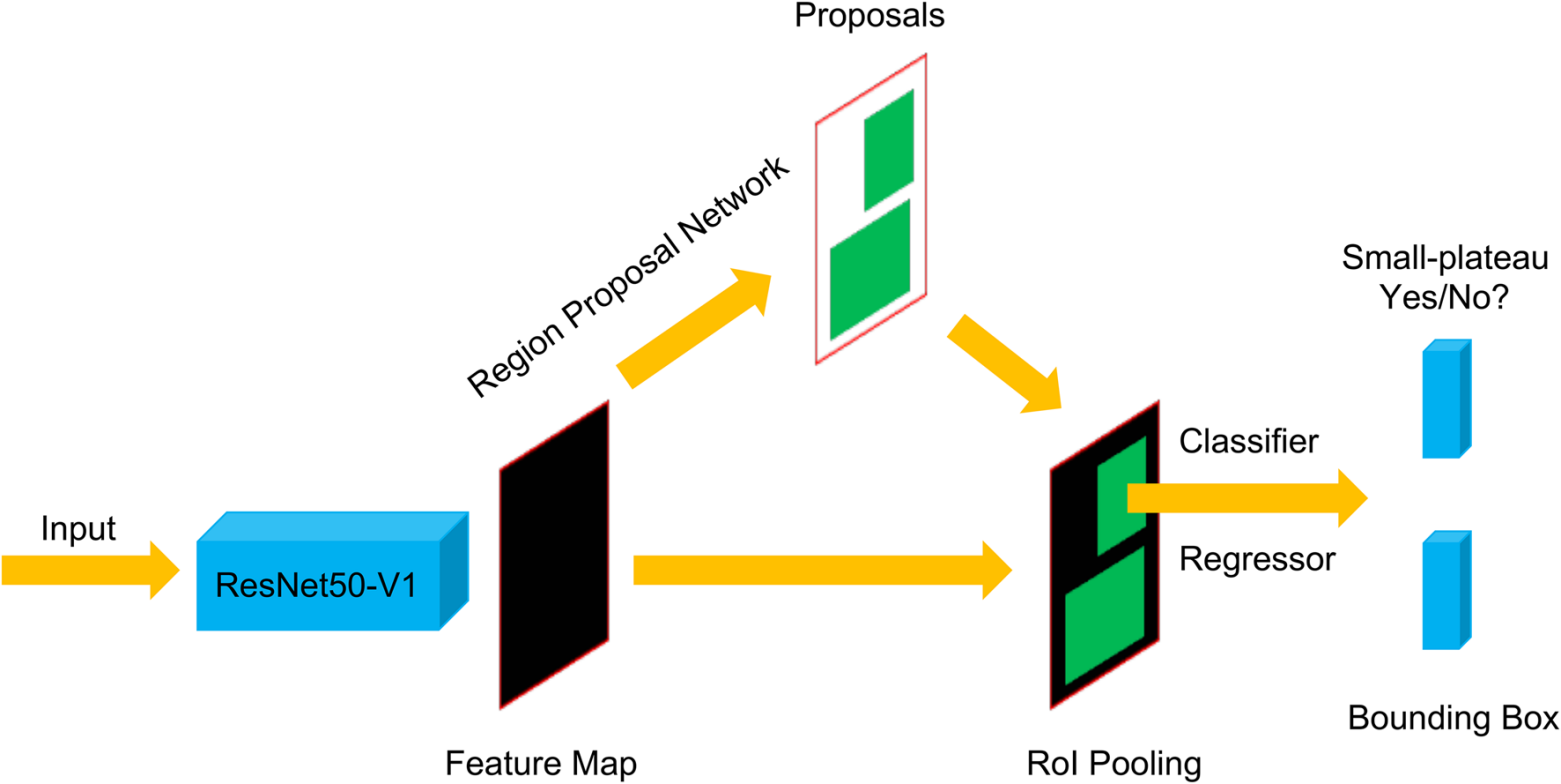
Architecture of SP-Net. When given an input image, the classic ResNet convolutional neural network was utilized to extract the feature map. Then a region proposal network would generate object bounds and objectness scores. Next, a RoI pooling layer would extract a feature vector from the feature map for each of the proposals. Each feature vector was fed into a series of fully connected layers that finally branch into a classifier and a regressor which output: (1) Whether an SP sign was detected; (2) Bounding box positions. RoI = region of interest; SP = small plateau; SP-Net = SP-network

As shown in Fig. 3, given an input image, the classic ResNet50 [17] convolutional neural network was utilized to extract the feature map. Then a region proposal network would generate object bounds and objectness scores, i.e., the probability of a specific sign occurs and its position, according to the feature map in a sliding window fashion. Next, a region of interest pooling layer would extract a feature vector from the feature map for each of the proposals. Each feature vector was fed into a series of fully connected layers that finally branch into a classifier and a regressor which output: (1) Whether an SP sign was detected; (2) Bounding box positions.

The model was firstly trained for 20,000 iterations with a learning rate of 1e-3 and then trained for another 10,000 iterations with a smaller learning rate of 1e-4. The batch size was set to 1 through all iterations. A stochastic gradient descent optimizer was utilized to train the model. Our training objective function was a multi-task loss following Fast R-CNN [23], which consisted of a classification loss and a regression loss for calculating the difference between model predictions and ground truth values.

### Statistical analysis

Count data were analyzed by using the χ^2^ or the Fischer exact test, as appropriate. Median comparisons were performed by using the Kruskal–Wallis test, or Mann–Whitney U test when there were two groups. Mean comparisons were performed by using the One-Way ANOVA or independent-samples T-test, as appropriate.

The deep learning model was implemented using TensorFlow 1.14.0 and trained on 4 Tesla K80 GPUs. Other statistical analyses were performed with SPSS version 26.

## Results

**Fig. 4:**
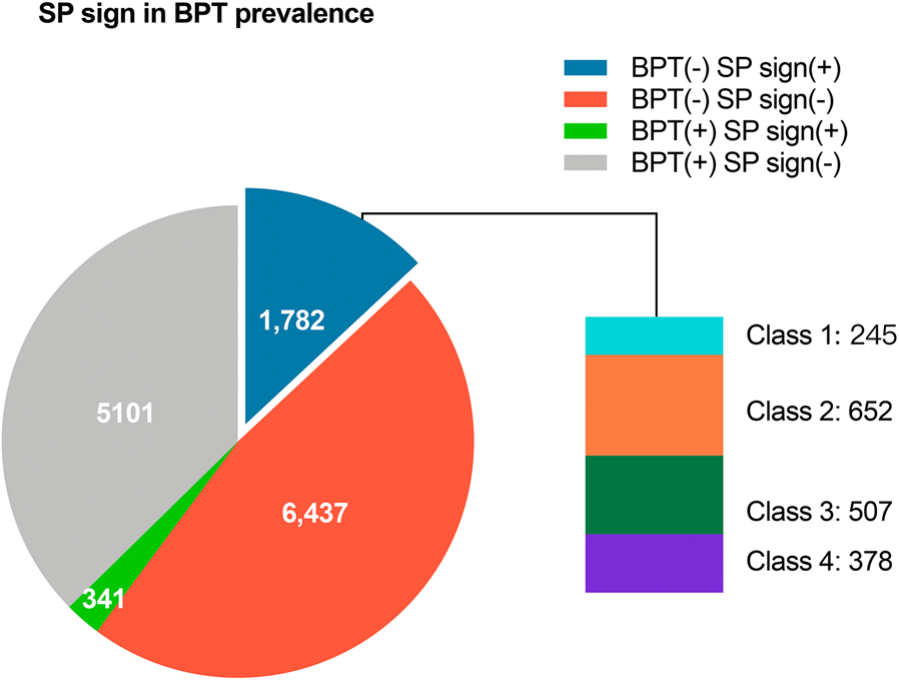
Prevalence of SP sign of all patients in BPTs. BPT (−) SP sign (+) was defined as patients with SP sign and had negative-BPTs; BPT (−) SP sign (−) was defined as patients without SP sign and had negative-BPTs; BPT (+) SP sign (+) was defined as patients with SP sign and had positive-BPTs; BPT (+) SP sign (−) was defined as patients without SP sign and had positive-BPTs. Classes were defined as follows: class 1 = (Vol A − Vol B) × 100/FVC ratio ≤ 10% in BPT (−); class 2 = (Vol A − Vol B) × 100/FVC ratio > 10% to ≤ 20% in BPT (−); class 3 = (Vol A − Vol B) × 100/FVC ratio > 20% to ≤ 30% in BPT (−); class 4 = (Vol A − Vol B) × 100/FVC ratio > 30% in BPT (−). BPTs = bronchoprovocation tests; Vol = volume; SP = small plateau

**Fig. 5:**
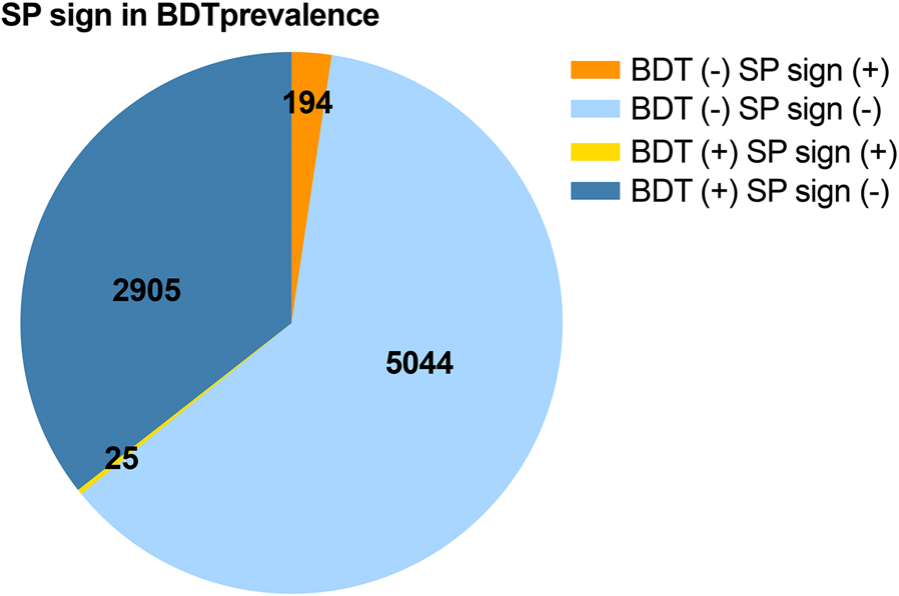
Prevalence of SP sign of all patients in BDTs. BDT (−) SP sign (+) was defined as patients with SP sign and had negative-BDTs; BDT (−) SP sign (−) was defined as patients without SP sign and had negative-BDTs; BDT (+) SP sign (+) was defined as patients with SP sign and had positive-BDTs; BDT (+) SP sign (−) was defined as patients without SP sign and had positive-BDTs. BDTs = bronchodilator tests; SP = small plateau

Of the 13,661 BPTs reviewed, 2,123 (15.5%) patients with SP sign were labelled. Of these, 1,782 (83.9%) were negative-BPT; 341 (16.1%) were positive-BPT (Fig. 4). Of a total 8,168 BDTs reviewed, there were 219 (2.7%) labeled as the SP sign. Of these, 194 (88.6%) were negative-BDT, 25 (11.4%) were positive- BDT (Fig. 5).

### Characteristic PFT parameters

SP sign was calculated mostly located on the 90–70% PEF and 18–40% FVC expiratory phase. Compared with positive-BPT, subjects with a negative-BPT had a more obvious SP sign which appeared to be wider and higher in the pre-challenge spirometry (both *P* < 0.0001) (Table 1). Most of the width of the sign of subjects with a positive-BPT narrowed or even disappeared in the post-BPT. On the contrary, subjects with a positive-BDT mostly without the sign in the prebronchodilator spirometry, the sign appeared in the post-bronchodilator maneuvers. (Table 1).

**Table 1:** Characteristic parameters

Parameter	SP sign					
	BPT (−) (N = 1782)	BPT (+) (N = 341)	*P* value	BDT (−) (N = 194)	BDT (+) (N = 25)	*P* value
Pre-BDT or BPT, %						
Vol A/FVC ratio	19.0 (16.1, 23.1)	19.6 (15.9, 23.2)	.468	17.5 (13.9, 21.8)	0.0 (0.0, 9.8)	< .0001
Flow A/PEF ratio	90.6 (86.2, 93.8)	87.6 (83.6, 92.1)	< .0001	88.6 (82.5, 93.1)	0.0 (0.0, 78.0)	< .0001
Vol B/FVC ratio	40.9 (32.5, 49.1)	37.6 (31.4, 44.5)	< .0001	30.2 (22.9, 36.8)	0.0 (0.0, 14.3)	< .0001
Flow B/PEF ratio	73.7 (64.9, 81.3)	73.5 (67.8, 79.9)	.404	78.3 (69.6, 85.2)	0.0 (0.0, 71.1)	< .0001
(Vol A − Vol B)/FVC ratio	19.9 (13.2, 28.4)	16.6 (11.0, 22.7)	< .0001	10.0 (6.3, 16.6)	0.0 (0.0, 3.3)	< .0001
(Flow A − Flow B)/PEF ratio	15.9 (10.1, 22.3)	12.4 (8.3, 18.1)	< .0001	8.7 (5.5, 13.8)	0.0 (0.0, 3.7)	< .0001
Post-BDT or BPT, %						
Vol A/FVC ratio	18.6 (15.2, 23.0)	0.0 (0.0, 10.2)	< .0001	17.5 (14.9, 22.1)	16.6 (14.9, 19.1)	.083
Flow A/PEF ratio	90.3 (85.2, 94.1)	0.0 (0.0, 68.7)	< .0001	89.2 (83.8, 93.4)	88.0 (83.9, 93.3)	.498
Vol B/FVC ratio	35.0 (26.6, 43.9)	0.0 (0.0, 15.6)	< .0001	33.8 (25.4, 40.3)	32.9 (25.2, 37.9)	.331
Flow B/PEF ratio	76.5 (66.4, 84.2)	0.0 (0.0, 56.9)	< .0001	76.2 (68.2, 83.0)	73.5 (69.2, 80.0)	.258
(Vol A − Vol B)/FVC ratio	14.1 (8.8, 22.4)	0.0 (0.0, 3.6)	< .0001	12.8 (8.5, 20.6)	15.8 (10.3, 20.2)	.226
(Flow A − Flow B)/PEF ratio	11.7 (6.8, 18.2)	0.0 (0.0, 2.5)	< .0001	11.5 (7.0, 16.8)	13.2 (8.7, 19.0)	.181

Data are presented as median values and quartiles unless otherwise noted. Statistical comparisons based on percent unless otherwise noted for characteristic parameters of small plateau sign specific values. *P* values < .05 were considered to represent a significant difference in prevalence between SP signs of the negative and positive BPT or BDT categories

BPT, bronchoprovocation test; BDT, bronchodilator test; SP, small plateau; Vol, volume; FVC, forced vital capacity; PEF, peak expiratory flow

Of the negative-BPT group, patients with SP sign were more often female and had a prevalence of obesity (BMI ≥ 30 kg/m^2^) (both, *P* < 0.0001) (Table 2). In pre-BPT and pre-BDT groups, compared patients without SP sign, patients with SP sign had higher median FVC, FEV_1_, FEF_50%_, FEF_75%_, and MMEF % predicted (all *P* < 0.0001); patients with the sign also had a higher median FEV_1_/FVC ratio (*P* = 0.009, *P* < 0.0001, *P* < 0.0001 and *P* < 0.0001, respectively) (Tables 2 and 3).

**Table 2:** PFT values of patients with pre-BPTs

Parameter	BPT (+)			BPT (−)		
	SP sign (+) (N = 341)	SP sign (−) (N = 5101)	*P* value	SP sign (+) (N = 1782)	SP sign (−) (N = 6437)	*P* value
Age, years	37 (24, 52)	21 (8, 48)	< .0001	39 (30, 51)	40 (30, 53)	.173
Sex, n (%)			< .0001			< .0001
Male	121 (35%)	2618 (51%)		714 (40%)	3162 (49%)	
Female	220 (65%)	2483 (49%)		1068 (60%)	3275 (51%)	
BMI, kg/m^2^	22.3 (19.6, 24.9)	20.1 (16.0, 23.6)	< .0001	22.6 (20.4, 24.8)	22.6 (20.1, 25.0)	.337
Obesity, n (%) (BMI ≥ 30 kg/m^2^)	13/341 (4%)	114/5101 (2%)	.062	54/1782 (3%)	90/6437 (1%)	< .0001
FVC % predicted, %	102.4 (94.9, 112.0)	98.8 (90.9, 107.3)	< .0001	103.0 (94.9, 112.0)	100.0 (91.0, 100.6)	< .0001
FEV_1_% predicted, %	97.5 (88.2, 105.8)	91.9 (82.1, 101.1)	< .0001	101.0 (93.4, 109.2)	97.0 (88.2, 106.0)	< .0001
FEV_1_/FVC ratio	0.80 (0.75, 0.84)	0.79 (0.72, 0.85)	.009	0.83 (0.79, 0.87)	0.81 (0.77, 0.86)	< .0001
PEF % predicted, %	95.5 (85.6, 105.5)	92.0 (81.4, 103.6)	< .0001	103.8 (93.0, 114.7)	103.6 (92.5, 115.2)	.824
FEF_50%_ % predicted, %	74.6 (60.2, 88.7)	63.1 (49.0, 80.0)	< .0001	86.3 (71.8, 103.3)	79.0 (63.2, 95.6)	< .0001
FEF_75%_ % predicted, %	55.8 (40.7, 71.0)	49.5 (35.6, 67.6)	< .0001	65.0 (50.7, 84.4)	62.0 (46.4, 82.1)	< .0001
MMEF % predicted, %	65.8 (52.6, 81.4)	59.2 (44.2, 76.8)	< .0001	77.5 (64.1, 92.4)	72.7 (57.5, 88.7)	< .0001

Data are presented as absolute numbers in case of frequencies, median values, and quartiles in case of continuous parameters. Statistical comparisons based on percent predicted (pp) unless otherwise noted for pulmonary function test (PFT) values. *P* values < .05 were considered to represent a significant difference in prevalence between SP signs of the negative and positive BPT categories

BMI, body mass index; FEV_1_, forced expiratory volume in 1 s; FEF_x%_, instantaneous forced expiratory flow when x% of the FVC has been expired; MMEF, maximal mid-expiratory flow

**Table 3:** PFT values of patients with pre-BDTs

Parameter	BDT (+)			BDT (−)		
	SP sign (+) (N = 25)	SP sign (−) (N = 2904)	*P* value	SP sign (+) (N = 194)	SP sign (−) (N = 5044)	*P* value
Age, years	35 (29, 57)	57.5 (45, 66)	< .0001	55 (45, 63)	62 (52, 69)	< .0001
Sex, n (%)			.014			< .0001
Male	12 (48%)	2047 (70%)		105 (54%)	3554 (70%)	
Female	13 (52%)	857 (30%)		89 (46%)	1490 (30%)	
BMI, kg/m^2^	22.5 (19.9, 24.2)	22.5 (19.8, 25)	.795	23.7 (21.4, 26.0)	22.2 (19.5, 24.8)	< .0001
Obesity, n (%) (BMI ≥ 30 kg/m^2^)	0/25 (0%)	96 (3%)	1.000	7/194 (4%)	127 (3%)	.347
FVC % predicted, %	92.8 (82.9, 100.5)	78.1 (65.1, 91.0)	< .0001	101.1 (88.5, 112.5)	80.0 (66.8, 94.4)	< .0001
FEV_1_% predicted, %	72.1 (63.9, 78.5)	51.0 (35.9, 64.6)	< .0001	88.4 (78.5, 97.0)	58.0 (40.0, 74.0)	< .0001
FEV_1_/FVC ratio	0.67 (0.62, 0.71)	0.52 (0.42, 0.63)	< .0001	0.73 (0.68, 0.77)	0.59 (0.44, 0.69)	< .0001
PEF % predicted, %	76.1 (69.5, 84.4)	52.6 (36.8, 69.4)	< .0001	90.9 (78.5, 102.7)	62.1 (42.0, 82.5)	< .0001
FEF_50%_ % predicted, %	38.5 (27.0, 47.3)	18.0 (10.3, 30.2)	< .0001	52.9 (42.9, 67.1)	23.8 (11.9, 40.8)	< .0001
FEF_75%_ % predicted, %	24.2 (18.5, 33.5)	15.5 (10.8, 23.0)	< .0001	31.3 (23.1, 43.0)	19.4 (12.6, 29.7)	< .0001
MMEF % predicted, %	30.9 (21.1, 40.6)	15.8 (9.7, 25.2)	< .0001	44.2 (34.4, 53.6)	20.5 (11.3, 34.9)	< .0001

Data are presented as absolute numbers in case of frequencies, median values, and quartiles in case of continuous parameters. Statistical comparisons based on percent predicted (pp) unless otherwise noted for pulmonary function test (PFT) values. *P* values < .05 were considered to represent a significant difference in prevalence between SP signs of the negative and positive BDT categories

PFT, pulmonary function test; BMI, body mass index; BDT, bronchodilator test; SP, small plateau; FVC, forced vital capacity; PEF, peak expiratory flow; FEV_1_, forced expiratory volume in 1 s; FEF_x%_, instantaneous forced expiratory flow when x% of the FVC has been expired; MMEF, maximal mid-expiratory flow

### Pre-PFT questionnaires

Patients with SP sign had higher rates of chronic cough in BPT groups (both *P* < 0.0001) (Table 4). In addition, patients with the sign were more likely to have evidence of wheezing in positive and/or negative BPT and BDT groups (*P* = 0.033, *P* < 0.0001, *P* = 0.011 and *P* = 0.014, respectively) (Tables 4 and 5).

**Table 4:** Pre-PFT questionnaires of BPTs

Parameter	BPT (+)		*P* value	BPT (−)		*P* value
	SP sign (+) (N = 205)	SP sign (−) (N = 3961)		SP sign (+) (N = 1124)	SP sign (−) (N = 4624)	
Smoking status, n (%)			.348			< .0001
Current or former smoker	23 (11%)	535 (14%)		155 (15%)	888 (19%)	
Never smoker	182 (89%)	3426 (86%)		969 (86%)	3753 (81%)	
Chronic cough, n (%)			< .0001			< .0001
Rare	49 (24%)	1428 (36%)		171 (15%)	1176 (25%)	
Sometimes	156 (76%)	2533 (64%)		947 (85%)	3448 (75%)	
Sputum production, n (%)	105/205 (51%)	1967/3961 (50%)	.663	568/1124 (51%)	2478/4624 (54%)	.066
Wheezing, n (%)	77/205 (38%)	1209/3961 (31%)	.033	342/1124 (30%)	865/4624 (19%)	< .0001
PFT indication, n (%)						
Cough	61/205 (30%)	1345/3961 (34%)	.360	663/1124 (59%)	2494/4624 (54%)	< .0001
COPD	2/205 (1%)	152/3961 (4%)	.034	64/1124 (6%)	237/4624 (5%)	.443
Asthma	162/205 (79%)	2098/3961 (53%)	< .0001	162/1124 (14%)	675/4624 (15%)	.875
Bronchiectasis	5/205 (2%)	26/3961 (1%)	.016	46/1124 (14%)	90/4624 (2%)	< .0001
ILD	0/205 (0%)	17/3961 (0%)	1.000	10/1124 (1%)	55/4624 (1%)	.394
Rhinitis/sinusitis	50/205 (24%)	46/3961 (1%)	< .0001	147/1124 (13%)	50/4624 (1%)	< .0001
Gastroesophageal reflux	2/205 (1%)	1/3961 (0.03%)	.007	15/1124 (1%)	3/4624 (0%)	< .0001
Pneumonia	10/205 (5%)	29/3961 (0.7%)	< .0001	58/1124 (5%)	102/4624 (2%)	< .0001
Shortness of breath	5/205 (2%)	65/3961 (2%)	.393	43/1124 (4%)	143/4624 (3%)	.213
Chest distress	4/205 (2%)	35/3961 (1%)	.123	29/1124 (3%)	121/4624 (3%)	.945
Others^a^	4/205 (2%)	147/3961 (4%)	1.000	7/1124 (1%)	654/4624 (14%)	< .0001

Data are presented as absolute numbers in the case of frequencies. *P* values < .05 were considered to represent a significant difference in prevalence between SP signs of the negative and positive BPT

PFT, pulmonary function test; COPD, chronic obstructive pulmonary disease; ILD, interstitial lung disease

^a^Indications include hypertension, coronary heart disease, diabetes, eosinophilic granulomatosis with polyangiitis, and studies without documented indication

**Table 5:** Pre-PFT questionnaires of BDTs

Parameter	BDT (+)			BDT (−)		
	SP sign (+) (N = 12)	SP sign (−) (N = 2235)	*P* value	SP sign (+) (N = 119)	SP sign (−) (N = 3728)	*P* value
Smoking status, n (%)			.111			.005
Current or former smoker	3 (25%)	1083 (48%)		45 (38%)	1929 (52%)	
Never smoker	9 (75%)	1172 (52%)		74 (62%)	1799 (48%)	
Chronic cough, n (%)			.338			.270
Rare	2 (17%)	625 (28%)		27 (23%)	1016 (27%)	
Sometimes	10 (83%)	1610 (72%)		92 (77%)	2712 (73%)	
Sputum production, n (%)	9/12 (75%)	1559/2235 (70%)	1.000	75/119 (63%)	2651/3728 (71%)	.056
Wheezing, n (%)	11/12 (92%)	1234/2235 (55%)	.011	68/119 (57%)	1703/3728 (46%)	.014
PFT indication, n (%)						
Cough	2/12 (17%)	262/2235 (12%)	.643	8/119 (7%)	328/3728 (9%)	.430
COPD	0/12 (0%)	809/2235 (34%)	.006	33/119 (28%)	1696/3728 (45%)	< .0001
Asthma	10/12 (83%)	905/2235 (40%)	.002	38/119 (32%)	720/3728 (19%)	< .0001
Bronchiectasis	0/12 (0%)	54/2235 (2%)	1.000	11/119 (9%)	293/3728 (8%)	.582
ILD	0/12 (0%)	12/2235 (1%)	1.000	3/119 (3%)	70/3728 (2%)	.493
Rhinitis/sinusitis	3/12 (25%)	18/2235 (1%)	< .0001	21/119 (18%)	39/3728 (1%)	< .0001
Gastroesophageal reflux	0/12 (0%)	1/2235 (0%)	1.000	3/119 (3%)	0/3728 (0%)	< .0001
Pneumonia	3/12 (25%)	56/2235 (3%)	.003	16/119 (13%)	210/3728 (6%)	< .0001
Shortness of breath	1/12 (8%)	114/2235 (5%)	.468	5/119 (4%)	176/3728 (5%)	.792
Chest distress	0/12 (0%)	9/2235 (0%)	1.000	2/119 (2%)	25/3728 (1%)	.203
Others^a^	0/12 (0%)	48/2235 (2%)	1.000	5/119 (4%)	171/3728 (5%)	.843

Data are presented as absolute numbers in the case of frequencies. *P* values < .05 were considered to represent a significant difference in prevalence between SP signs of the negative and positive BDT categories

COPD, chronic obstructive pulmonary disease; ILD, interstitial lung disease

^a^Indications include hypertension, coronary heart disease, diabetes, eosinophilic granulomatosis with polyangiitis, and studies without documented indication

In negative-BPT groups, patients with the sign were more likely to have PFT indications of cough, bronchiectasis, rhinitis (sinusitis), gastroesophageal reflux and pneumonia (all *P* < 0.0001) (Table 4). In positive-BDT groups, PFT indications of asthma, rhinitis (sinusitis), and pneumonia had higher rates, but COPD had a lower rate within the sign (*P* = 0.002, *P* < 0.0001, *P* = 0.003, and *P* = 0.006, respectively); patients with the sign had higher rates of PFT indications of asthma, rhinitis (sinusitis), gastroesophageal reflux and pneumonia in the negative-BDT group (all *P* < 0.0001), on the contrary, a lower rate of PFT indication of COPD (*P* < 0.0001) (Table 5).

### Baseline characteristics of the SP sign classes

In the width-stratified classes, of 1,782 subjects, there were 245 (13.7%) with class 1, 652 (36.6%) with class 2, 507 (28.5%) with class 3, and 378 (21.2%) subjects with class 4 (Fig. 4). Compared with class 1, subjects with class 4 were more often never smoker (*P* = 0.005) and with higher median FEV_1_% predicted and FEV_1_/FVC ratio (*P* = 0.007 and *P* = 0.003, respectively). Patients with class 1 had a higher rate of PFT indication of COPD compared to class 4 (*P* < 0.0001) (Table 6). In the height-stratified classes, there were 439 (24.6%) with class 1, 759 (42.6%) with class 2, 474 (26.6%) with class 3, and 110 (6.2%) subjects with class 4. Compared with class 2, class 4 were with higher median FEV_1_% predicted (*P* = 0.009). However, patients with class 1 had a higher FEV_1_/FVC ratio than other classes (*P* < 0.0001) (Table 7).

**Table 6:** Baseline characteristics of the SP width-stratified classes in negative pre-BPTs

Parameter	SP sign of negative pre-BPTs				
	(Vol A − Vol B) × 100/FVC ratio (≤ 10%) (N = 50)	(Vol A − Vol B) × 100/FVC ratio (> 10% to ≤ 20%) (N = 50)	(Vol A − Vol B) × 100/FVC ratio (> 20% to ≤ 30%) (N = 50)	(Vol A − Vol B) × 100/FVC ratio (> 30%) (N = 50)	*P* value
Age, years	39.1 ± 2.2	41.9 ± 2.2	40.6 ± 1.8	37.7 ± 2.0	.797
Sex, n (%)					.002
Male	31 (62%)^c,d^	25 (50%)	14 (28%)^a^	17 (34%)^a^	
Female	19 (38%)^c,d^	25 (50%)	36 (72%)^a^	33 (66%)^a^	
BMI, kg/m^2^	22.4 (19.8, 24.3)	22.4 (19.9, 24.5)	22.4 (20.2, 25.2)	21.3 (20.2, 24.6)	.884
Obesity, n (%) (BMI ≥ 30 kg/m^2^)	0/50 (0%)	2/50 (4%)	2/50 (4%)	2/50 (4%)	.727
Underweight, n (%) (BMI < 18.5 kg/m^2^)	5/50 (10%)	5/50 (10%)	5/50 (10%)	5/50 (10%)	1.000
Smoking status, n (%)					.005
Current or former smoker	17 (34%)^d^	7 (14%)	5 (10%)	6 (12%)^a^	
Never smoker	33 (66%)^d^	43 (86%)	45 (90%)	44 (88%)^a^	
FVC % predicted, %	102.5 (95.3, 108.2)	101.7 (92.4, 111.1)	104.1 (98.1, 113.3)	106.3 (95.1, 115.4)	.200
FEV_1_% predicted, %	97.7 (91.9, 103.3)^d^	98.0 (91.9, 108.0)	102.6 (96.0, 112.5)	105.2 (98.1, 111.4)^a^	.007
FEV_1_/FVC ratio	0.81 ± 0.01^d^	0.82 ± 0.01	0.83 ± 0.01	0.85 ± 0.01^a^	.003
Chronic cough, n (%)					< .0001
Rare	6 (12%)^d^	11 (22%)	5 (10%)	26 (52%)^a^	
Sometimes	44 (88%)^d^	39 (78%)	45 (90%)	24 (48%)^a^	
Sputum production, n (%)	24/50 (48%)	24/50 (48%)	22/50 (44%)	24/50 (48%)	.971
Chest tightness, n (%)	0/50 (0%)	0/50 (0%)	0/50 (0%)	1/50 (2%)	1.000
Wheezing, n (%)	15/50 (30%)	14/50 (28%)	8/50 (16%)	16/50 (32%)	.264
PFT indication, n (%)					
Asthma	7 (14%)	8 (16%)	6 (12%)	2 (4%)	.253
COPD	10 (20%)^d^	3 (6%)	0 (0%)	1 (2%)^a^	< .0001
ILD	0 (0%)	2 (4%)	0 (0%)	0 (0%)	.246
Pulmonary nodule/neoplasia	1 (2%)	2 (4%)	1 (2%)	0 (0%)	.903
Cough	26 (52%)	28 (56%)	28 (56%)	29 (58%)	.943
Shortness of breath	1 (2%)	1 (2%)	2 (4%)	3 (6%)	.839
Rhinitis/sinusitis	5 (10%)	2 (4%)	5 (10%)	2 (4%)	.477
Other^e^	0 (0%)^d^	4 (8%)	8 (16%)	13 (26%)^a^	.001

Data are presented as absolute numbers (percentages) in case of frequencies, median values, and quartiles in case of continuous parameters unless otherwise noted. Statistical comparisons based on percent predicted (pp) unless otherwise noted for pulmonary function test (PFT) values

^a^*P* < 0.05; (Vol A − Vol B) × 100/FVC ratio ≤ 10% versus the remaining groups

^b^*P* < 0.05; (Vol A − Vol B) × 100/FVC ratio > 10% to ≤ 20% versus the remaining groups

^c^*P* < 0.05; (Vol A − Vol B) × 100/FVC ratio > 20% to ≤ 30% versus the remaining groups

^d^*P* < 0.05; (Vol A − Vol B) × 100/FVC ratio > 30% versus the remaining groups

^e^Indications include bronchiectasis, chest pain, pleural effusion, sleep disorder, and studies without documented indication

**Table 7:** Baseline characteristics of the SP height-stratified classes in negative pre-BPTs

Parameter	SP sign of negative pre-BPTs				
	(Flow A − Flow B) × 100/PEF ratio (≤ 10%) (N = 50)	(Flow A − Flow B) × 100/PEF ratio (> 10% to ≤ 20%) (N = 50)	(Flow A − Flow B) × 100/PEF ratio (> 20% to ≤ 30%) (N = 50)	(Flow A − Flow B) × 100/PEF ratio (> 30%) (N = 50)	*P* value
Age, years	39.1 ± 2.2	41.9 ± 2.2	40.6 ± 1.8	37.7 ± 2.0	.119
Sex, n (%)					.262
Male	29 (58%)	16 (32%)	26 (52%)	13 (26%)	
Female	21 (42%)	34 (68%)	24 (48%)	37 (74%)	
BMI, kg/m^2^	23.5 (21.1, 26.3)	23.1 (20.5, 25.3)	22.3 (19.8, 24.9)	21.3 (20.2, 24.6)	.156
FVC % predicted, %	104.9 ± 2.0	103.1 ± 1.8	103.6 ± 1.80	107.1 ± 1.8	.408
FEV_1_% predicted, %	100.9 ± 1.6	99.1 ± 1.6^d^	101.6 ± 1.4	106.4 ± 1.7^b^	.009
FEV_1_/FVC ratio	1.01 (0.94, 1.09)^b,c,d^	0.81 (0.79, 0.84)^a^	0.84 (0.80, 0.87)^a^	0.85 (0.81, 0.88)^a^	.000

Data are presented as absolute numbers (percentages) in case of frequencies, median values, and quartiles in case of continuous parameters unless otherwise noted. Statistical comparisons based on percent predicted (pp) unless otherwise noted for pulmonary function test (PFT) values

^a^*P* < 0.05; (Flow A − Flow B) × 100/PEF ratio ≤ 10% versus the remaining groups

^b^*P *< 0.05; (Flow A − Flow B) × 100/PEF ratio > 10% to ≤ 20% versus the remaining groups

^c^*P* < 0.05; (Flow A − Flow B) × 100/PEF ratio > 20% to ≤ 30% versus the remaining groups

^d^*P* < 0.05; (Flow A − Flow B) × 100/PEF ratio > 30% versus the remaining groups

### Laryngoscopy findings

**Fig. 6:**
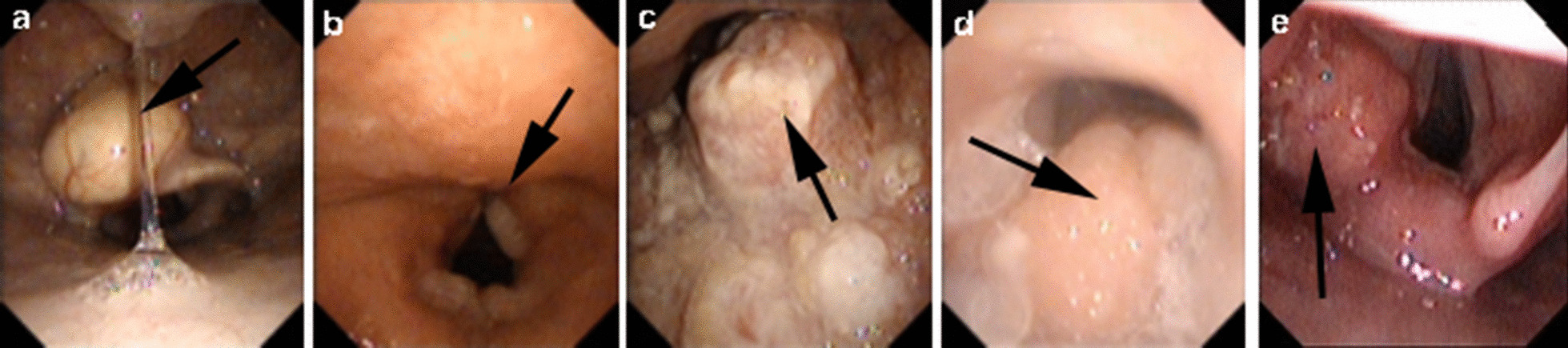
Examples of the laryngoscopy findings. **a** Epiglottic cyst; **b** vocal cord nodule; **c** tonsil mass, **d** hypertrophy of tonsils/adenoids; **e** supraglottic laryngeal cancer. The location indicated by the black arrow is the lesion

A total of 48 patients who underwent the laryngoscopy completed the FS tests. Of these, 32 (66.7%) patients without SP sign and 16 (33.3%) patients with the sign. Patients with SP sign were less likely to have laryngoscopy evidence of chronic pharyngitis or normal upper airway compared with those without the sign (*P* = 0.038) (Table 8). They showed a higher percentage of upper airway abnormalities. Figure 6 shows examples of these abnormalities. Further analysis of the degree of upper airway stenosis [24] due to these findings, we found that the degree of stenosis of patients with SP sign were all within 0∼25%, however, patients without SP sign showed different degrees of stenosis, which distributed in ∼25%, 50%, 75%, and 90% (Table 9).

**Table 8:** Laryngoscopy findings associated with the SP sign

Parameter	SP sign (+) (n = 16)	SP sign (−) (n = 32)	*P* value
Age, years	44.2 ± 3.1	50.0 ± 3.0	.223
Sex, n (%)			.147
Male	7 (44%)	21 (66%)	
Female	9 (56%)	11 (34%)	
BMI, kg/m^2^	25.0 ± 1.0	22.5 ± 0.8	.060
Smoking status, n (%)			.404
Current or former smoker	5 (31%)	14 (44%)	
Never smoker	11 (69%)	18 (56%)	
Laryngoscopy finding, n (%)			
Epiglottis cyst	2 (13%)	0 (0%)	.106
Vocal cord/throat nodule/tumor	4 (25%)	7 (22%)	1.000
Hypertrophy of tonsils/adenoids	4 (25%)	3 (9%)	.195
Chronic pharyngitis/normal	6 (37%)	22 (69%)	.038

Data are presented as absolute numbers (percentages) in case of frequencies unless otherwise noted

**Table 9:** Precision data summary

Degree of stenosis	SP sign (+) (N = 10)	SP sign (−) (N = 10)
∼ 25%	10	5
50%	0	2
75%	0	1
90%	0	2

Data are presented as absolute numbers in the case of frequencies

SP, small plateau

### Automated recognition

**Fig. 7:**
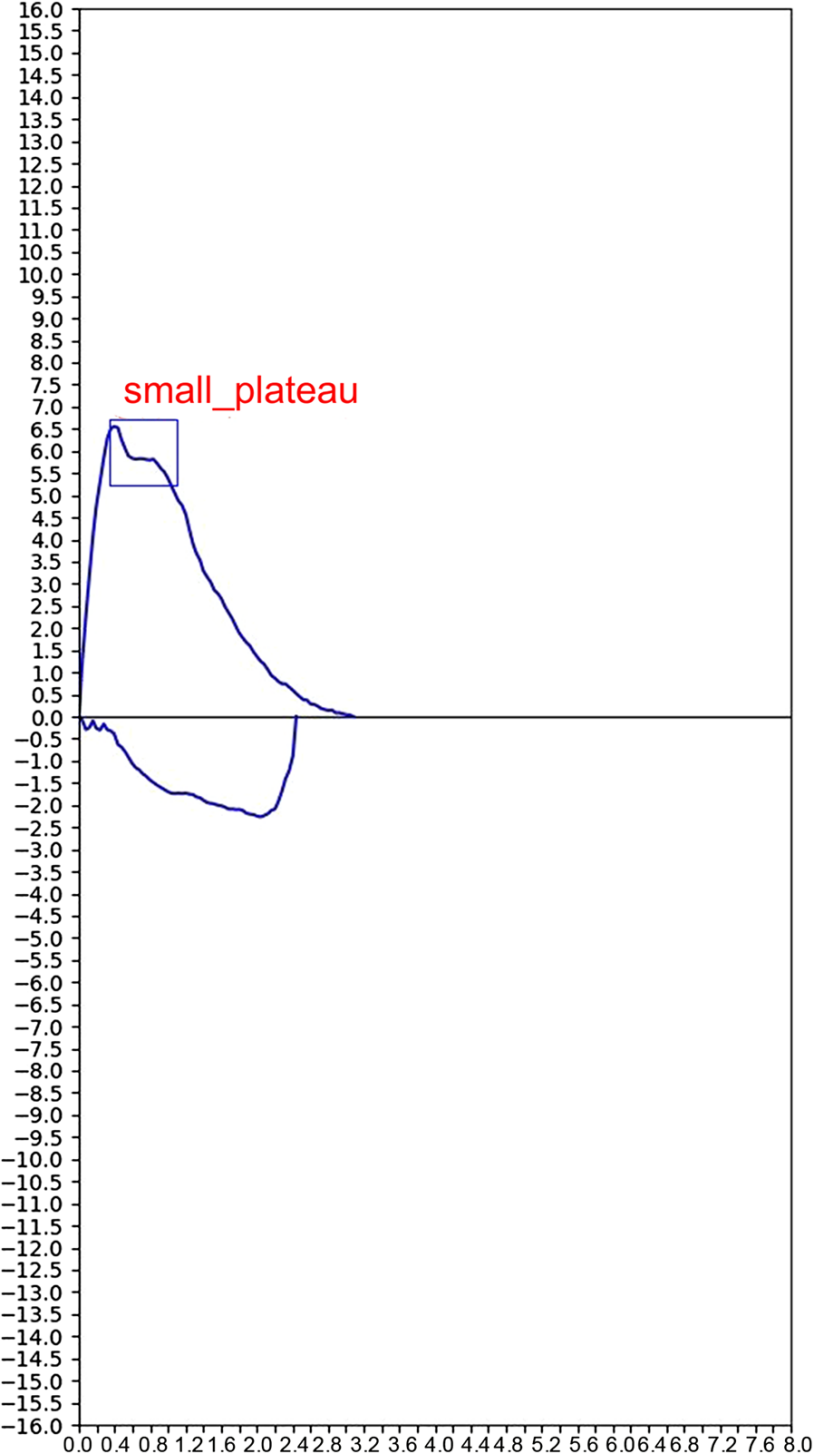
Model output. The SP-Net drew a red bounding box to represent the detected position of the SP sign and also generated corresponding annotations. SP = small plateau; SP-Net = SP-network

**Fig. 8:**
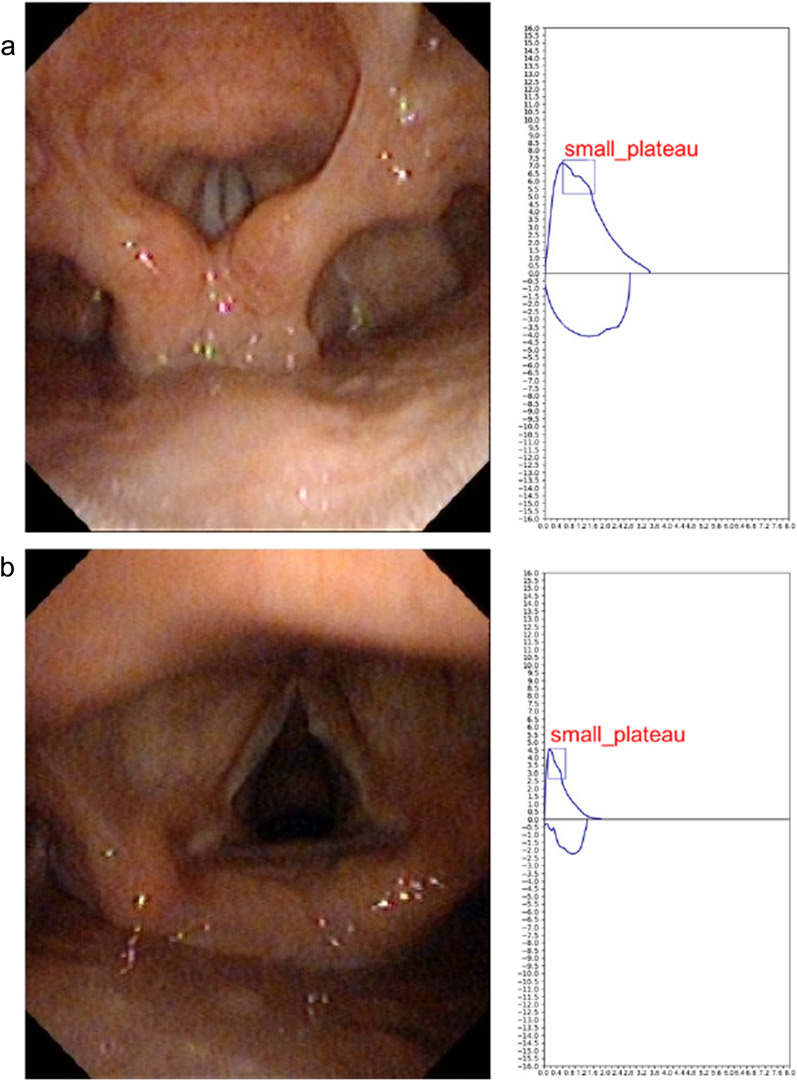
Examples of the laryngoscopy findings and corresponding model outputs of the SP sign. **a** Normal; **b** vocal cord polyp. SP = small plateau

An example of the output of our model is shown in Fig. 7. After running the inference, our model would draw a red bounding box to represent the detected position of the SP sign and also generated corresponding annotations. Figure 8 visualizes two case studies consisting of laryngoscopy findings and corresponding model outputs. In both normal (A) and vocal cord polyp (B) cases, our SP-Net successfully detected the SP sign.

**Fig. 9:**
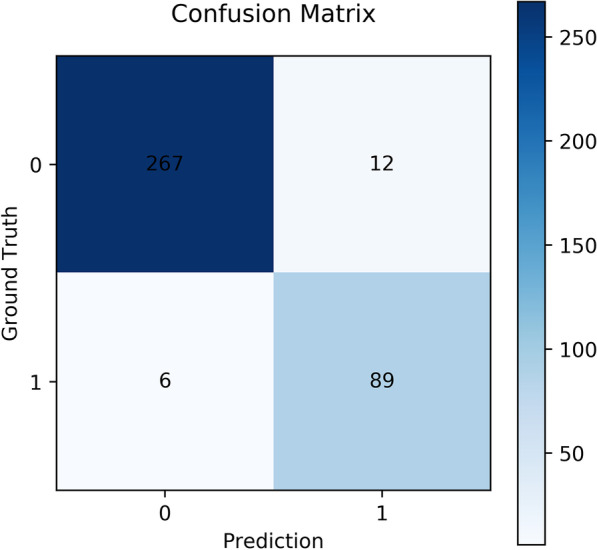
Confusion matrix. 0: subjects without SP sign, 1: subjects with SP sign. SP = small plateau

The performance of our proposed deep learning model was evaluated on the test set using five metrics. The evaluation showed that our model achieved 95.2% accuracy, 93.7% sensitivity, 95.7% specificity, 88.1% positive predictive value, and 97.8% negative predictive value (Table 10 and Fig. 9).

**Table 10:** Performance evaluation (N = 374 records)

	Accuracy	Sensitivity	Specificity	PPV	NPV
SP-Net	95.2% (93.0–97.4)	93.7% (91.2–96.2)	95.7% (93.6–97.8)	88.1% (84.8–91.4)	97.8% (96.3–99.3)

Data are presented as mean and 95% CI

SP, small plateau; SP-Net, SP-Network; PPV, positive predictive value; NPV, negative predictive value

## Discussion

Visual inspection of the flow-volume curve is a simple and valuable approach to diagnose and evaluate diseases, such as obstructive sleep apnea, upper airway obstruction, and unilateral main-stem bronchial obstruction [25–28]. The SP sign is a common but previously not widely recognized configuration of the curve that adds value to classify airway responsiveness. It has been defined as the presence of “a small plateau of the early phase of expiratory flow on the flow-volume curve” [5].

The current study found that patients with SP sign were mostly negative-BPTs. Since a positive-BPT usually means patient with AHR, when spirometry showing an SP sign, that might indicate the patient is less likely to have AHR. This result was in agreement with the previous studies [5, 6]. The prevalence of the SP sign in BDTs was only 2.7%, of them, the rate of negative-BDTs was 88.6%, this outcome indicated patients with SP sign had lower airway reversibility. Additionally, we noted the disappearing or narrowing of the width of the sign in the post-BPT maneuvers of patients with a positive-BPT, while the SP sign presented in the post-BDT maneuvers of patients with a positive-BDT. As indicated above, these findings supported the SP sign less on the flow-volume curves with severe ventilatory defects, or the sign could be masked because of the methacholine challenge-induced decline of PFT values. The median FVC, FEV_1_, FEF_50%_, FEF_75%_, and MMEF % predicted were all higher in patients with SP sign, whereas Li et al. [5] found no significant difference values between patients with or without SP sign using BPT data. In theory, our findings were also confirmed in BDT data. Moreover, a recent study has evaluated baseline spirometry variables as markers for AHR, they found that all baseline spirometry parameters were significantly lower in the positive AHR group. In their study, FEF_50%_ was proved to be a negative predictor for AHR [12]. In line with our study, SP sign also thought to be a negative marker, patients with SP sign presented higher FEF_50%_ % predicted.

Further analysis of pre-BPTs questionnaires, we demonstrated that patients with SP sign were more likely to have PFT indications, including cough, rhinitis (sinusitis), and gastroesophageal reflux. On the contrary, the indications due to asthma and COPD were not significantly different compared to those without the sign. In theory, these diseases have been more often with symptoms like cough, sputum, and abnormal sensation of the throat, which were significantly associated with upper airway abnormalities.

The higher median FEV_1_% predicted and FEV_1_/FVC ratio in SP sign width-stratified class 4 compared to 1, demonstrated the sign presented more often in subjects with a normal-to-mild ventilatory defect. In addition, class 4 had lower rates of chronic cough and PFT indication of COPD, but had higher rates of PFT indications of bronchiectasis, chest pain, pleural effusion, and sleep disorder. These findings indicated that with the increase of the width of the sign, types of diseases were more complex and the symptoms were more diverse.

In 48 patients with laryngoscopy findings, we observed patients with SP sign had a lower prevalence of chronic pharyngitis or normal finding. They were more likely to have evidence of upper airway stenosis due to epiglottis cyst, hypertrophy of tonsils, vocal cord nodule, and throat tumor, etc. However, the degree of stenosis in patients with SP sign was mostly 0∼25%. In line with this tendency, Li et al. [5] observed the endoscopy findings of nine subjects with a negative-BPT and SP sign, they found that these subjects were excluded from severe central airway stenosis. These findings suggested that the SP sign may be an indicator to identify upper airway disorders.

Because the SP sign presented a good indicator to classify patients who were less likely to have AHR and the degree of severity of spirometric abnormality, we developed an SP-Net to automatically recognize it with an accuracy of 95.2%. The confusion matrix has shown that the SP-Net was sensitive (93.7%) when assessed on the test set (N = 374 cases). Six positive cases were wrongly detected negative because some curves have a concave shape at the same location of the SP sign. These shapes also appeared repeatedly in the different curves of one person. However, we did not know their physiological mechanism, fortunately, they were not common. A previous study proposed a neural network to detect upper airway obstruction caused by goiter using the flow-volume curve [29]. Artificial intelligence also helped with the interpretation and quality control of PFTs, together with the diagnosis of chronic respiratory diseases [14, 30, 31]. To the best of our knowledge, we were the first to use deep learning models for automatic detection of SP sign. Our object detection model not only could classify the image type, but could simultaneously locate the position of the SP sign. This additional feature could serve as interpretable guidance for the doctors to analyze and understand based on what visual cues the model classified a sample into the category of SP sign. It  was very promising that our proposed deep learning algorithms could serve as a simple tool to aid SP sign recognition in primary care. In the future, the model can be integrated into the software of lung function equipment, more tasks of recognition of specific configuration of flow-volume curves, such as a saw-tooth sign, upper airway obstruction, and unilateral main bronchial stenosis will be developed. It will help alert the non-specialists to the potential presence of some disorders which sometimes be misdiagnosed.

A major limitation of this study was acquired data retrospectively, which could not observe symptoms of patients during BPTs. Although the SP sign was mostly present in patients with a negative-BPT, they were more likely to have evidence of symptoms due to upper airway abnormalities. The symptoms during BPTs will need to be observed in future work. Another drawback of our study was a lack of results of AHR in patients with laryngoscopy findings. They were only assigned to complete a baseline spirometry test, but not a BPT, since performing a BPT takes more time and may bring a bad experience to the subject. In addition, the level of inter-rater variability of authors when labelled the spirometry files could not be calculated. Because no one has re-labelled files that others’ have already labelled. Finally, we only developed an approach to recognition SP sign using SP-Net, future studies should incorporate clinical manifestations, PFT indices, and laryngoscopy findings to assist clinicians in the classification of healthy people, patients with asthma and COPD, as well as the prediction of airway responsiveness.

## Conclusions

We analyzed the prevalence, clinical, and lung function characteristics of the SP sign. The sign mostly presented in patients with normal-to-mild ventilatory defect and a negative-BPT. Patients with SP sign were less likely to have AHR and severe ventilatory defects. We propose the SP sign as an indicator to perform the classification of patients with asthma who are mostly with AHR and patients with COPD who are mostly with obvious airway obstruction. The application of the sign is ideal for primary care that cannot carry out BPTs. Therefore, we developed SP-Net to automate the recognition of it. Additional studies will need to further define the sign, including its airway dynamics and physiology mechanisms.

## Data Availability

The datasets used and/or analyzed during the current study are available from the corresponding author on reasonable request.

## Abbreviations

AHR: Airway hyperresponsiveness
BDT: Bronchodilator test
CV: Computer vision
BPT: Bronchoprovocation test
COPD: Chronic obstructive pulmonary disease
FEF_x%_: Instantaneous forced expiratory flow when x% of the FVC has been expired
FS: Forced spirometry
MMEF: Maximal mid-expiratory flow
PFT: Pulmonary function test
SP: Small plateau
SP-Net: SP-Network
